# More Than Just a Click: A Five-Step Guide to Mobile Medical Photography for Amateurs

**DOI:** 10.7759/cureus.89668

**Published:** 2025-08-09

**Authors:** NJASS Jayasuriya, BM Munasinghe, KAL Ravihari, SAAAN Sirimanna, MKDHV Jayalath, GGSY Weerasundara

**Affiliations:** 1 Anaesthesiology, Base Hospital, Thambuththegama, Thambuththegama, LKA; 2 Anaesthesiology and Critical Care, Base Hospital, Thambuththegama, Thambuththegama, LKA; 3 Surgery, Base Hospital, Thambuththegama, Thambuththegama, LKA

**Keywords:** five steps, images, medical, mobile, photography

## Abstract

Medical photography is crucial in modern medicine, and this article offers a five-step framework to help healthcare workers take clear, professional clinical photos. It is widely used globally for diagnosis, documentation, education, and publication. Smartphones have made capturing medical images easier, even without formal training, but quality varies without proper guidance. The editorial also covers ethical issues such as consent and patient privacy to protect confidentiality. Following best practices and ethics can improve image quality while maintaining patient trust.

## Editorial

Background

Medical photography is a vital part of modern healthcare, supporting diverse clinical, documentation, educational, and research activities. With ongoing advances in mobile phone technology, mobile phone cameras now match the image quality of professional cameras [1]. This development has made mobile phones crucial in medical photography, enabling healthcare professionals with a smartphone to instantly capture high-resolution clinical images without needing formal photography training. Medical photography encompasses more than just capturing clinical findings in live patients. It includes a wide range of subjects, such as non-biological objects (such as medical instruments, prosthetic devices, surgical tools, and medical documents) and biological specimens (such as excised organs and pathological samples). Each category presents unique challenges related to composition, exposure, background, and scale. For example, a well-taken image of a skin lesion can reveal features essential for diagnosis. In specialized fields such as wound care and dermatology, mobile photographs have become standard in patient records and referral processes [2]. They improve patient care by enabling peer consultations, monitoring treatment progress, and supporting patient follow-up. Nonetheless, increased access to medical photography does not automatically mean one is an expert. Amateur photos often have issues, such as improper orientation, poor lighting, or ethical oversights, which can reduce their clinical or publication usefulness. Low-quality images may delay diagnosis and treatment or lead to misinterpretation [3]. Incorrect photographic methods can also result in significant errors and misrepresentations of surgical results [4]. The authors, based on their experience, propose a five-step guide to help amateurs improve their medical photography, producing clearer, more valuable, and more accurate images.

Five-step approach to take compelling medical images

**Figure 1:**
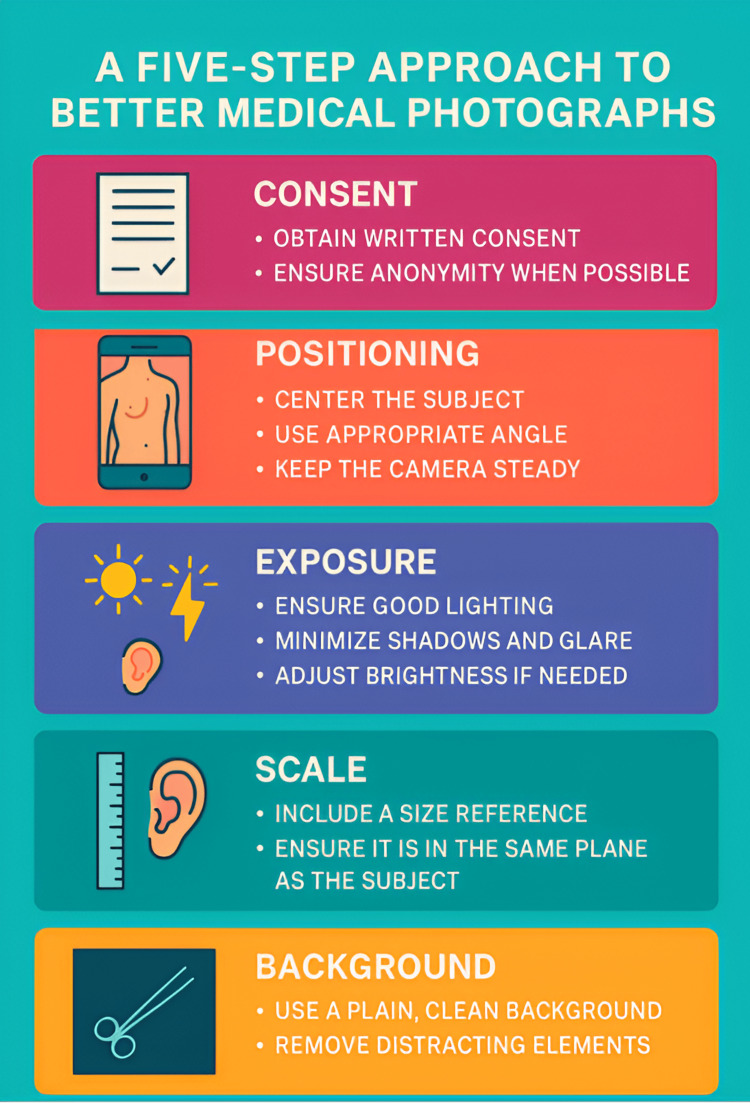
Five-step guide to optimal medical photography

To ensure high-quality images, healthcare providers can follow a five-step framework (Figure 1).

This framework combines core concepts in photography into a few practical steps that any provider can apply when using a smartphone or any other imaging device. These steps include consent, positioning, exposure, background, and scale.

Consent

Patient consent is not just a formality, but a crucial step in the process of taking a medical photograph [5]. It is a demonstration of respect for the patient's autonomy and privacy. One must always obtain informed consent before taking any photo of a patient. Written consent is ideal, especially for publication purposes. It is essential to explain to the patient why the image is being taken (e.g., for a medical record, consultation, or publication). Make sure to respect privacy. Only take photos when they serve a clear clinical, educational, or research purpose. Avoid casual photography without ethical justification [6]. An institutional consent form for photography can be used to document consent [7]. It is essential to note that patient confidentiality must always be maintained, even with informed consent. This means de-identifying images when possible (omitting the face or any identifiable marks if not clinically relevant) and handling the images as protected health information. Ensuring consent first fulfils ethical obligations and enables patients to trust and feel comfortable.

Positioning

Correct positioning of the subject and the camera is fundamental to capturing an informative image. In a human subject, position the area of interest in an anatomically standard position whenever feasible. The camera must be held perpendicular to the subject and aimed straight at the location of interest to avoid distortion. For a photograph to be effective, the subject must be centered in the frame, particularly for specimens and non-biological objects. Try to leave equal space between the subject and the photo border. Include relevant anatomical landmarks for context. For example, when photographing a skin lesion, take a photo from a standard distance that shows the lesion in relation to an anatomical landmark, and then take a closer photo focusing on the lesion's details [3]. Good positioning also involves taking shots from multiple angles when needed. For instance, a surgical wound or a swollen joint may benefit from oblique or lateral views to depict depth and contour [8]. When photographing a removed organ or specimen, place it on a flat surface to avoid distortion. For instruments or devices, align the subject symmetrically and ensure clear visibility to identify features or structural components.

Exposure and Lighting

Exposure in photography is the amount of light that reaches the camera sensor, determining how bright or dark a photo appears [9]. Incorrect exposure can make an image overly bright or dark, sacrificing the correct colors and details. Although a smartphone is typically programmed to adjust the exposure of an image when tapped to focus automatically, it may still struggle when the lighting conditions are poor [10]. Thus, ensuring adequate lighting is available dramatically improves the image quality. Take pictures in a well-lit environment or near a window with ample indirect natural light [11]. Overhead examination lamps or additional LED lights can provide even illumination of the subject. If lighting is insufficient, a smartphone's flash can be used. However, direct flash can create glare, harsh shadows, or incorrect colors [12]. In that case, the intensity of the flash can be adjusted in most phones to achieve better results. Positioning a light source at an angle to the subject can reduce harsh shadows and glare. If the external light source is excessively bright, a white surface, such as a wall or a sheet of paper, can reflect the light onto the subject, potentially causing unwanted shadows. Tap on the area of interest on your smartphone's screen to focus and auto-adjust the exposure before taking the image.

Background

The background of a medical photograph should be plain, distraction-free, and contrasting to highlight the subject [13]. A neutral, solid background (such as a blue or green drape or a white wall) provides good contrast. Remove any clutter, personal items, or extraneous objects from the field before taking the photo. The goal is to avoid confusing the viewer with irrelevant details. The subject should immediately draw the eye. Moreover, a consistent background across a series of photos helps compare images over time, eliminating background variability as a factor. If photographing a specimen or an excised organ, place it on a clean, plain tray of a contrasting color to make edges and details stand out. Ensure that sterile fields are not compromised during the photographing process.

Scale

Including a scale or a reference object in the photograph provides an immediate sense of size, which is crucial for medical interpretation. A common practice is placing a ruler or standardized measuring device adjacent to the photographed subject [14]. Ensure the scale is placed on the same plane as the subject, clearly visible but not covering any vital detail. If a standard scale is unavailable, even a coin or a known object can serve as an improvised scale. A medical ruler with high contrast markings is preferable for accuracy. In addition to a measurement scale, one can use anatomical landmarks as references for less precise size. For example, a lesion photographed on the hand, foot, or face can be measured in size by comparing it to known anatomical landmarks, such as fingers, the ear, or the eye.

**Figure 2:**
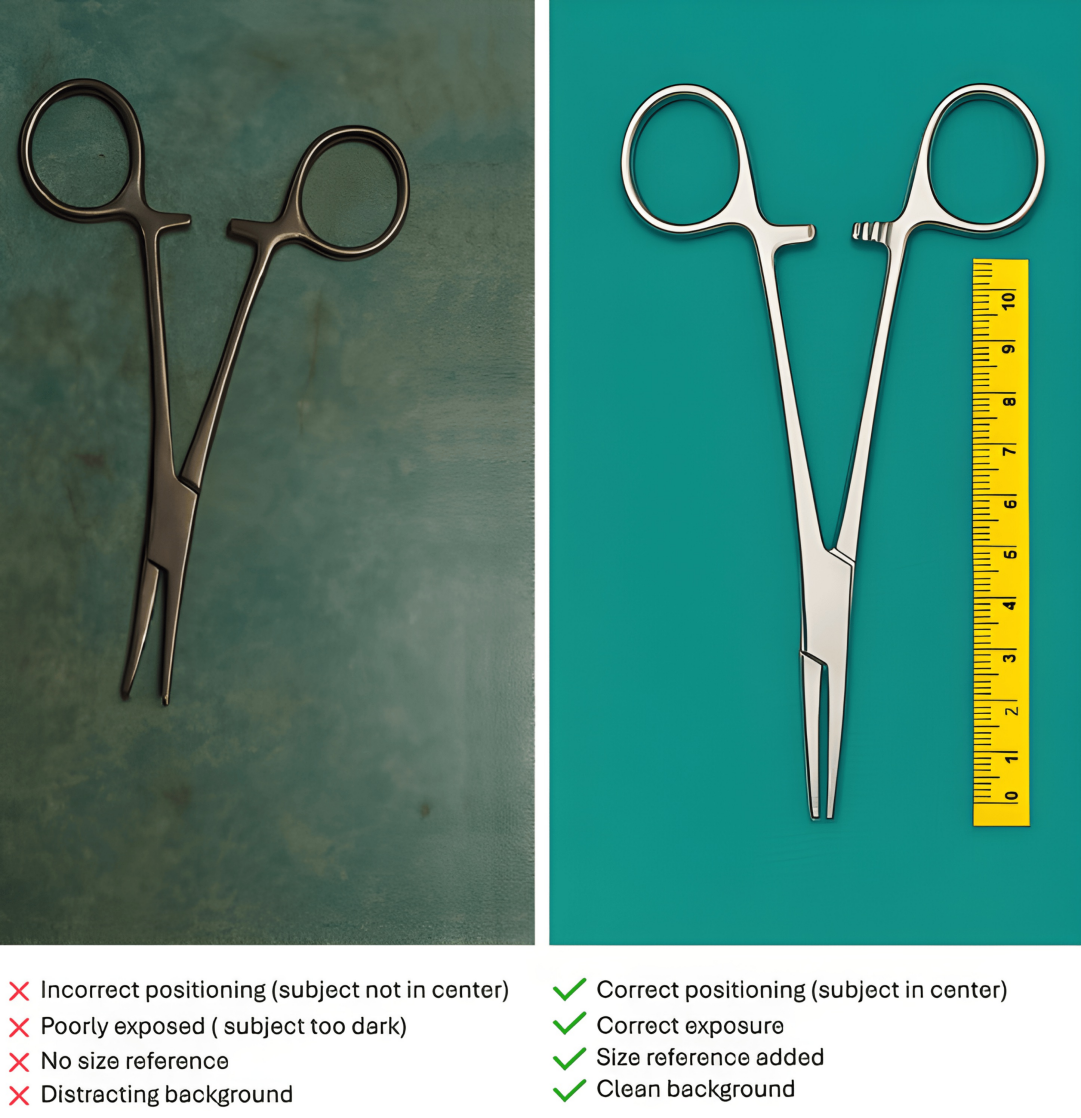
Comparison of the quality of photographs in different techniques Image: needle holder Image on the right obtained using a Samsung Galaxy S24 Ultra phone Light source: reflected surgical table light, distance: 30 cm

These simple steps can significantly improve image quality with minimal effort. As illustrated in Figure 2, a subject appears noticeably more transparent and better contextualized when photographed using these principles.

Additional best practices

Take Multiple Shots

Capture multiple photos of the same subject whenever possible. Take an overview and a close-up, and consider different angles or lighting. This increases the likelihood of getting at least one optimal image and provides choices for documentation. Redundant photos can be culled later, but critical details lost due to a single poor photo cannot be recovered.

Use Optimal Equipment Settings

Always use the smartphone's rear camera (which typically has the highest resolution) rather than the front camera. Set the camera to the highest resolution and quality available. Avoid using digital zoom, as it can reduce image quality. Instead, move physically closer to the subject (within the minimum focusing distance) to prevent focus issues and distortion, or crop the high-resolution image later if needed. Turn off filters or portrait modes that blur or alter parts of the image, as the goal is a clinically accurate photo, not an artistic one.

Maintain Stability

Camera shake is a common cause of blurriness. Hold the phone with steady hands, braced against your body or a surface. A small tripod or even resting the phone on a stack of books or a counter can help. Many newer smartphones have optical image stabilization, which helps when the phone is held correctly. However, mechanical stabilization (such as a tripod) beats hand-holding for absolute clarity, especially in low light [15].

Consider Accessories

In some cases, inexpensive accessories can improve results. Clip-on macro lenses can help capture fine details of small objects (such as sutures and dermatological lesions) without using digital zoom. External lights or ring flashes attached to the phone can provide more uniform lighting in dark environments. Ensure any accessory used is compatible and does not introduce artefacts (e.g., some attachments might cause vignetting or color changes if not aligned properly).

After-Capture Review and Storage

Once images are taken, review them on a larger screen if possible. What appears acceptable on a phone display may reveal focus issues or poor exposure when viewed on a computer monitor. If the image will be used in a publication or presentation, minimal post-processing is advised [16]. It is acceptable to adjust brightness or crop for focus, but avoid manipulation that could alter the clinical content. Always retain the original file. Use a secure method to transfer the photo to the patient's electronic health record or a secure storage, and then delete it from the personal device if policy requires. Importantly, do not share clinical photos via social media or standard messaging apps; use encrypted, approved channels to consult or refer if needed [17].

Ethical and privacy considerations

Medical photography must maintain a balance between the importance of the image and the respect for patient rights and privacy [18]. In addition to obtaining consent (step 1 above), clinicians are advised to adhere to institutional and legal guidelines concerning the management of patient images. All photographs of patients constitute part of the medical record and should be regarded as confidential health information. It is recommended to turn off cloud backups on smartphones or utilize specialized hospital-approved camera applications that encrypt images and upload them directly to secure servers. Such measures minimize the risk of a privacy breach in cases of device loss or hacking. Many healthcare institutions, particularly in high-resource settings, designate specific smart devices exclusively for medical photography within each ward or clinic to address concerns related to privacy, security, and patient comfort [19]. Utilizing a device solely for clinical documentation purposes reduces the likelihood of data breaches and promotes patient trust and adherence. Patients are more inclined to consent to photography when they understand that their images will remain within the clinical system rather than on a medical practitioner's device [20]. Various regions impose specific regulations, such as the Health Insurance Portability and Accountability Act (HIPAA) in the United States, which impose significant penalties for the unauthorized disclosure of patient images [21]. Consequently, de-identification of images serves as a vital ethical safeguard. It is imperative to preserve the dignity of the patient. Avoid capturing images that could be perceived as sensational or excessively graphic, unless such images are explicitly intended for educational purposes and obtained with proper consent. It is essential to recognize that, in a moral sense, the patient owns their image; healthcare providers act as stewards of these images for medical purposes [22]. By respecting privacy, obtaining informed consent, and employing secure handling procedures, healthcare professionals can incorporate photography into patient care in a manner that fosters trust and complies with professional standards.​ Recent practical tools, including automated face-blurring applications and encrypted "photo lockers," also facilitate effective de-identification of patient images.

Potential limitations and barriers

Reliance on smartphone functionality or applicability in resource-limited environments may restrict the universal adoption of medical photography. Similarly, security and privacy concerns encompass data breaches, insecure storage, the absence of integration with medical records, and challenges in obtaining consent. Furthermore, issues with image quality and precision may arise, including color fidelity, bias in automated analysis, limited depth of field, and lack of control. These factors can render the technique unsuitable for standardized photography and hinder its integration into electronic health records. Additional barriers include insufficient training and resources, as well as regional policies that may prohibit the use of mobile devices during medical photography.

Conclusion

Medical photography is vital in clinical practice, research, and education. With smartphones widely available, even non-professionals can capture quality images by following key principles. Clinicians can improve clarity, relevance, and ethics by considering consent, positioning, exposure, background, and scale. Using dedicated devices or apps helps protect patient privacy and ensure compliance. As medical imaging becomes more integrated into daily care, standardized, responsible photography is a clinical and professional duty. AI-based quality checks offer promising ways to improve medical imaging further.
